# Lifestyle and Clinical Factors in a Nationwide Stage III and IV Renal Cell Carcinoma Study

**DOI:** 10.3390/cancers15184488

**Published:** 2023-09-09

**Authors:** Nessn Azawi, Freja Ejlebaek Ebbestad, Naomi Nadler, Karina Sif Soendergaard Mosholt, Sofie Staal Axelsen, Louise Geertsen, Jane Christensen, Niels Viggo Jensen, Niels Fristrup, Lars Lund, Frede Donskov, Susanne Oksbjerg Dalton

**Affiliations:** 1 Department of Urology, Zealand University Hospital, Sygehusvej 10, 4000 Roskilde, Denmark; naon@regionsjaelland.dk; 2 Institute for Clinical Medicine, University of Copenhagen, Noerregade 10, 1165 Copenhagen, Denmark; sanne@cancer.dk; 3 Danish Cancer Institute, Strandboulevarden 49, 2100 Copenhagen, Denmark; freja.ejlebaek.ebbestad@regionh.dk (F.E.E.); jane@cancer.dk (J.C.); 4 Department of Urology, Rigshospital, Blegdamsvej 9, 2100 Copenhagen, Denmark; karina.sif.soendergaard.mosholt@regionh.dk; 5 Department of Oncology, Aarhus University Hospital, Palle Juul-Jensens Blvd. 161, 8200 Aarhus, Denmark; s.axelsen@rn.dk (S.S.A.); niels.fristrup@rm.dk (N.F.); 6 Department of Urology, Odense University Hospital, J. B. Winsløws Vej 4, 5000 Odense, Denmark; louise.geertsen@rsyd.dk (L.G.); lars.lund@rsyd.dk (L.L.); 7 Department of Oncology, Odense University Hospital, J. B. Winsløws Vej 4, 5000 Odense, Denmark; niels.viggo.jensen@rsyd.dk; 8 Department of Oncology, Southern Denmark University Hospital, Esbjerg, Finsensgade 35, 6700 Esbjerg, Denmark; fdonskov@health.sdu.dk; 9 Department of Clinical Oncology & Palliative Care, Zealand University Hospital, Rådmandsengen 5, 4400 Næstved, Denmark

**Keywords:** advanced stage renal cell carcinoma, lifestyle-related factors, patient-related risk factors, multidisciplinary team discussion, patient outcomes

## Abstract

**Simple Summary:**

Our research focused on understanding how specific patient and clinical factors, present at the diagnosis of advanced renal cell carcinoma (RCC), influence the mortality and recurrence of the disease. We studied patients with advanced RCC from a national Danish database and monitored them over time. Our findings showed that having a positive surgical margin, synchronous metastasis, and poor health status were linked with a higher chance of death and recurrence of the disease. Interestingly, for non-ccRCC patients, having a multidisciplinary team (MDT) discussion during diagnosis was found to lower the risk of death. This suggests that individual clinical details play more significant roles in RCC outcomes than lifestyle factors. Moreover, including MDT discussions in treatment plans may benefit patients.

**Abstract:**

Background: The aim was to investigate whether patient-related or clinical risk factors present at the diagnosis of advanced stage renal cell carcinoma (RCC) had an impact on the overall mortality, cancer-specific mortality, and recurrence risk in a national cohort. Methods: Patients registered with stage III and IV RCC in the Danish Renal Cancer Database (DaRenCa) in 2014–2016 were included in the study and followed up until recurrence or death. We conducted a Cox Proportional Hazard Model to examine the association between several variables and the development of RCC. These variables included BMI, hypertension, smoking status, symptoms at diagnosis, performance status, multidisciplinary team (MDT) discussion, surgical margin, and primary metastasis. Separate analyses were performed for cc-RCC and non-ccRCC patients. Results: In our cohort of 929 patients, 424 individuals died from RCC during the follow-up period, with a median follow-up time of 4.1 (95% CI: 0.8–5.0) years for ccRCC and 2.0 (95% CI: 0.1–5.0) years for non-ccRCC. A multivariate analysis demonstrated that a positive surgical margin (HR 1.53 and 1.43), synchronous metastasis (HR 2.06 and 3.23), and poor performance status (HR 4.73 and 5.27) were significantly associated with a decreased 5-year overall and cancer-specific survival, respectively. Furthermore, a positive surgical margin was associated with a higher risk of recurrence in ccRCC. MDT discussion was found to reduce mortality risk in non-ccRCC. Conclusion: Clinical- and disease-related variables have a greater impact on RCC mortality and recurrence than the selected lifestyle-related factors. The inclusion of MDT discussion in the diagnosis and management of advanced RCC should be further evaluated for its potential to improve patient outcomes.

## 1. Background

The incidence of renal cell carcinoma (RCC) has increased in recent years, particularly in Western countries, due to the aging population and increasing detection of incidental findings [1,2]. While patients diagnosed with early-stage RCC (stages I and II) have a favourable prognosis, those with advanced RCC (stages III and IV), approximately 15% of which present with metastasis at diagnosis, face a considerably worse outcome [3]. Clinical and pathological factors, such as TNM stage, Fuhrman grade, tumour size, tumour necrosis, and sarcomatoid differentiation, are known to play roles in prognosis and have been used to develop predictive models for survival and recurrence [4].

However, the impact of patient-related factors, such as lifestyle and general health, on prognosis has received less attention. Nevertheless, evidence suggests that factors such as hypertension [5], a poor performance status [6], and the presence of symptoms at diagnosis [5,6] may also play roles in survival. Despite the absence of metastasis at diagnosis, 20–30% of patients still develop recurrence within three years, highlighting the potential role of non-clinical factors in determining risk [3].

This study aims to examine the effect of patient-related and clinical factors on overall and cancer-specific mortality (RQ1) and recurrence (RQ2). Additionally, we will investigate the impact of time to recurrence and the treatment of recurrences on overall and cancer-specific mortality (RQ3).

## 2. Methods

Data were collected from the Danish Renal Cancer Database (DaRenCa) and medical records for patients diagnosed with stage III and IV renal cell carcinoma between 1 January 2014 and 31 December 2016. Approval and permissions were obtained from the Danish Patient Safety Authority (3-3013-2902/1) and the study is in accordance with the regulations set by the Danish Data Protection Agency (REG-041-2021). The data collection was performed between May 2020 and December 2021.

In addition to the information obtained from DaRenCa, we also utilized data from several other relevant national registries, including the Civil Registration System, the Danish National Patient Registry, the Danish National Pathology Registry, and the Danish Pathology Data Bank [7,8,9,10,11].

### 2.1. Exposures

#### Patient-Related Factors

Based on the information from the medical records, we defined the following patient-related factors as body mass index (BMI kg/m^2^) at diagnosis; hypertension (indicated by one or more active prescriptions for antihypertensive medication); smoking status as current, former, or never smoker; the presence of symptoms at diagnosis (pain, haematuria, weight loss, or other); and performance status categorized as 0, 1–2, or 3–4 using the Eastern Cooperative Oncology Group Performance status.

### 2.2. Clinical Characteristics and Treatment

The histology of RCC was categorized as clear cell or non-clear cell, T-stage was assigned according to the 2009 TNM classification [12], N-stage was assigned based on malignant lymph nodes in a histological examination, and M-stage was assigned based on pathological or radiological evidence of metastasis at diagnosis or within 120 days of diagnosis. We then defined stage III or IV based on T-stage, N-stage, and M-stage. The Leibovich score was calculated according to the Leibovich score 2003 [13], and a positive surgical margin was defined based on the final histological report. For treatment, we defined interaction with a multidisciplinary team (MDT) before surgery (yes/no), cytoreductive nephrectomy, and lymphadenectomy (yes/no), and the treatment of recurrence was categorized as surgical, oncological, combined, or none.

### 2.3. Outcomes

We defined the following events as outcomes: death of all causes, cancer-specific death, recurrence based on the information from medical records and computerized tomography scan reports, and death after recurrence.

### 2.4. Statistical Methods

Risk time was defined from the date of diagnosis until an event of interest or the last follow-up on 31 December 2021. The relationship between exposures of interest and outcomes of interest was evaluated using univariate and multivariate regression models. The Cox proportional hazards model was used to assess the effect of each exposure on overall death and cancer-specific death after one year and five years, as well as the effect of each exposure on recurrence. The models were adjusted for relevant covariates, including demographic information, clinical characteristics, patient health status, and treatment factors. The assumption of proportional hazards was tested using Schoenfeld residuals and the assumption of linearity of the continuous variables was tested by including squared and cubic variables in the Cox proportional hazard model. We took steps to mitigate the risk of immortal time bias by utilizing a time-dependent covariate analysis in the Cox Proportional Hazards Model.

The relationship between time to recurrence and death was evaluated using univariate and multivariate regression models. The Cox proportional hazards model was used to assess the effect of time to recurrence on overall death and cancer-specific death. The models were adjusted for relevant covariates, including demographic information, clinical characteristics, patient health status, and treatment factors. The assumption of proportional hazards was tested using Schoenfeld residuals and the assumption of linearity of the continuous variables was tested by including squared and cubic variables in the Cox model.

Multiple imputations were performed to handle missing data, which accounted for 13% (127 out of 929) of the study population. The method used for imputation was fully conditional specification, which was performed separately for clear cell RCC (ccRCC) and non-clear cell RCC (non-ccRCC) [14]. Body mass index (BMI), Fuhrman grade (only for ccRCC), T-stage, tumour size, smoking status, and performance status were imputed. These variables were imputed in their uncategorized form and later categorized after the imputation. The Leibovich score was calculated after the imputation and before the Cox proportional hazards model. All the variables included in the analysis, as well as the variables of operation status (yes/no), operation type, outcome, and follow-up time, were included in the imputation. In total, 50 imputations were calculated with 500 iterations. The results were combined using Rubin’s rules and reported as hazard ratios (HR) with 95% confidence intervals (CI).

**Figure 1:**
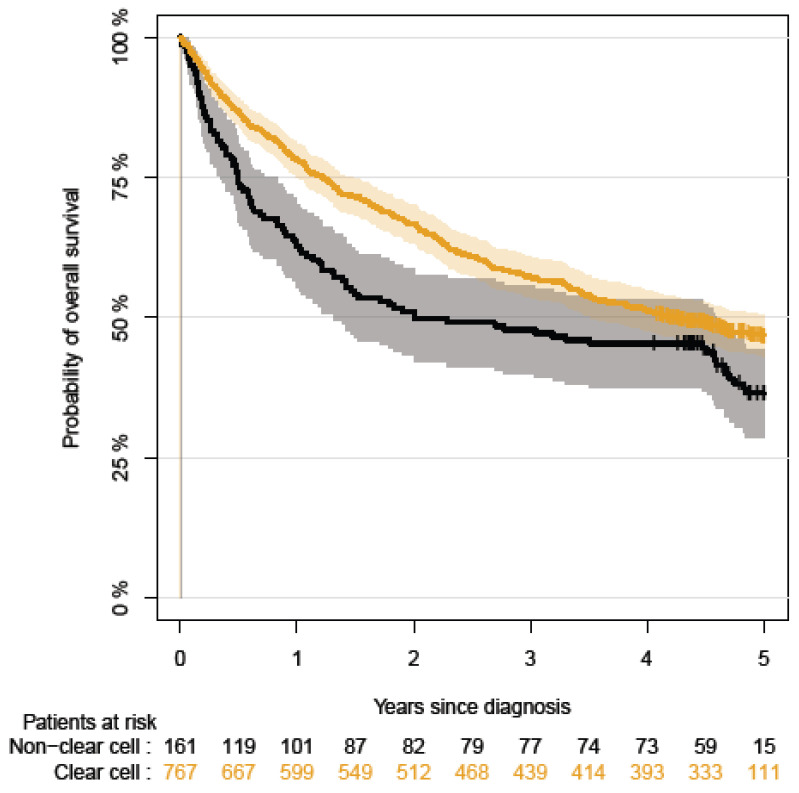
The five-year overall survival rates for patients with stage III and IV for non-ccRCC.

**Figure 2:**
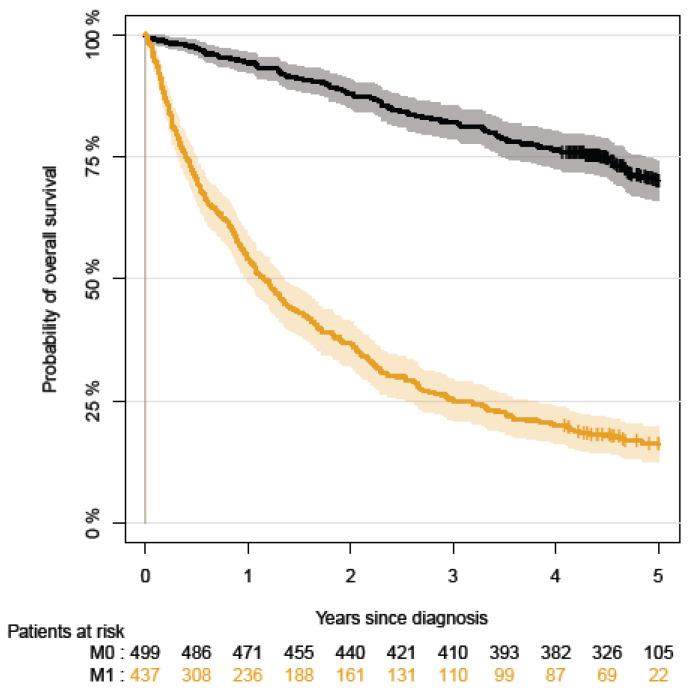
The five-year overall survival rates for patients with primary metastatic disease versus non-metastatic RCC.

**Figure 3:**
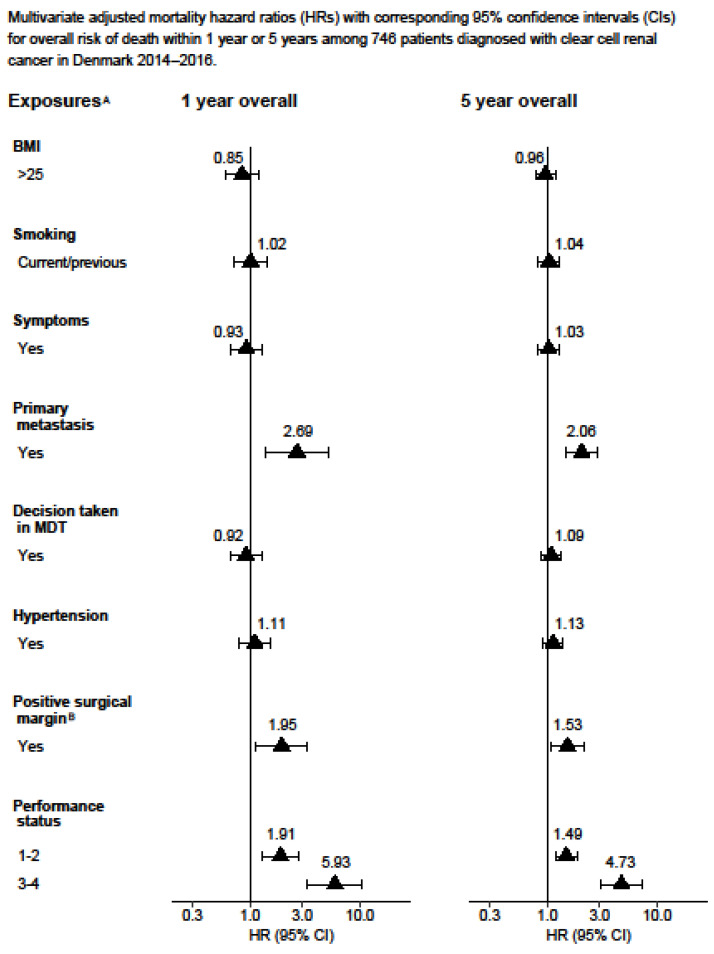
Multivariate adjusted mortality hazard ratios (HRs) with corresponding 95% confidence intervals (CIs) for overall risk of death within 1 year or 5 years among 746 patients diagnosed with clear cell renal cancer in Denmark 2014–2016. ^A^ Adjusted for age, gender, Leibovich score, sarcomatoid, smoking, hypertension, performance status, decision taken in MDT, debulking, and lymphadenectomy. ^B^ Also adjusted for partial or radical nephrectomy in model 1.

**Figure 4:**
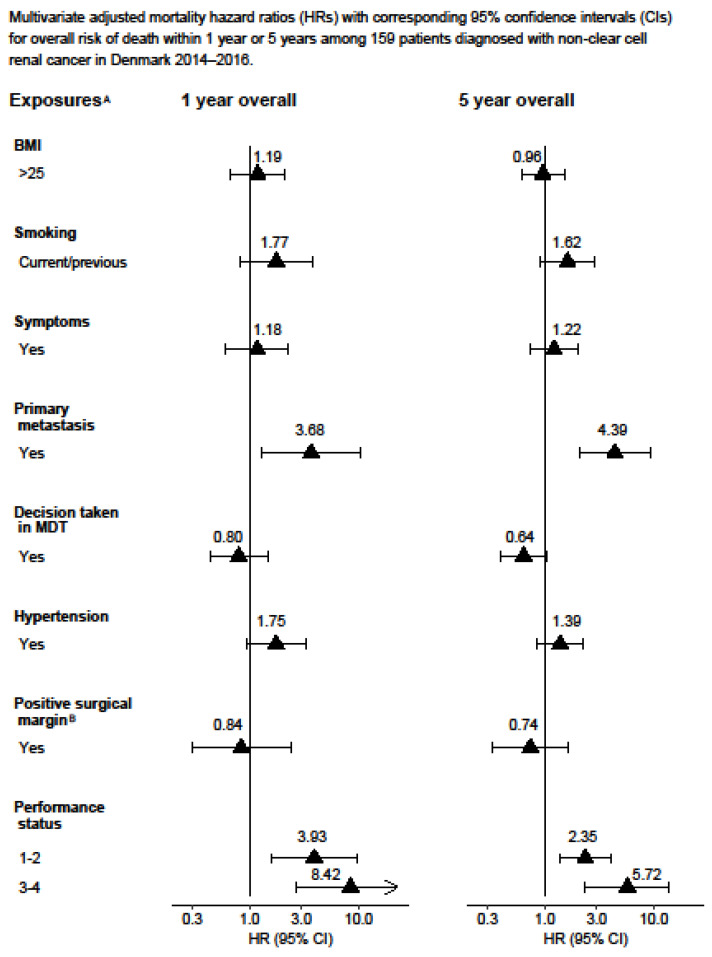
Multivariate adjusted mortality hazard ratios (HRs) with corresponding 95% confidence intervals (CIs) for overall risk of death within 1 year or 5 years among 159 patients diagnosed with non-clear cell renal cancer in Denmark 2014–2016. ^A^ Adjusted for age, T-stage, N-stage, M-stage, size of tumour, hypertension, performance status, and debulking. ^B^ Also adjusted for partial or radical nephrectomy.

**Figure 5:**
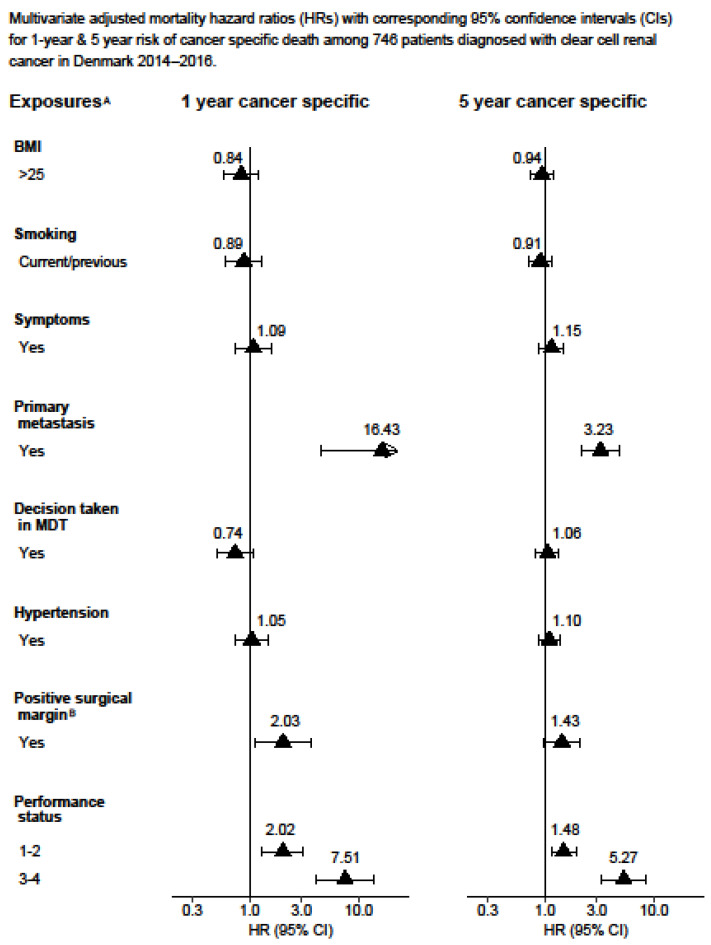
Multivariate adjusted mortality hazard ratios (HRs) with corresponding 95% confidence intervals (CIs) for 1-year and 5-year risk of cancer-specific death among 746 patients diagnosed with clear cell renal cancer in Denmark 2014–2016. ^A^ Model 3: Adjusted for age, gender, Leibovich score, sarcomatoid, smoking, hypertension, performance status, decision taken in MDT, debulking, and lymphadenectomy. ^B^ Also adjusted for partial or radical nephrectomy.

**Figure 6:**
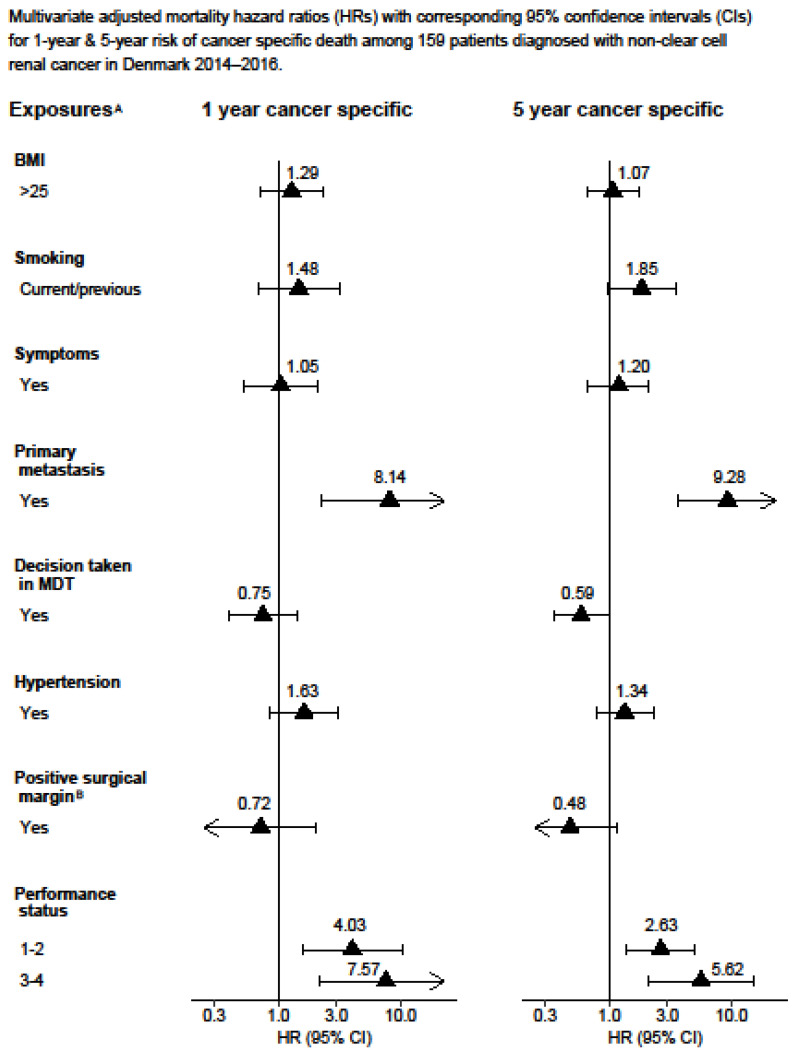
Multivariate adjusted mortality hazard ratios (HRs) with corresponding 95% confidence intervals (CIs)for 1-year and 5-year risk of cancer-specific death among 159 patients diagnosed with non-clear cell renal cancer in Denmark 2014–2016. ^A^ Adjusted for age, T-stage, N-stage, M-stage, size of tumour, sarcomatoid, hypertension, performance status, and debulking. ^B^ Adjusted for partial or radical nephrectomy.

The complete-case Cox proportional hazards model results can be found in the Supplementary Tables S1–S8. The statistical analysis was performed using Stata version 16.1. Figure 1 and Figure 2 display the Kaplan–Meier curves created using R version 4.1.2 and the packages “ggplot2”, “patchwork”, and “prodlim”. Meanwhile, Figure 3, Figure 4, Figure 5 and Figure 6 show other graphical presentations.

## 3. Results

### 3.1. Study Population Characteristics

A total of 929 patients diagnosed with stage III (53%) or stage IV (47%) renal cell carcinoma were included in the study. Table 1 displays the demographic and lifestyle characteristics of the study population. Out of the 929 patients, 525 (55%) died from any cause and, among these, 424 (45%) died from renal cell carcinoma. In total, 610 (66%) patients underwent radical nephrectomy and 73 (8%) underwent partial nephrectomy, with 81 (13%) and 13 (18%) patients having a positive surgical margin, respectively. At the time of diagnosis, 517 (56%) patients had hypertension. Some 296 (32%) patients underwent cytoreductive nephrectomy. During the follow-up, 170/494 patients (34%) experienced a recurrence of RCC, for which 31 of these patients (18%) received surgical treatment. The results obtained from the multiple imputations are presented in Figure 3, Figure 4, Figure 5 and Figure 6, whereas the results from the complete-case analysis can be found in Supplementary Tables S1, S2, and S4. In the complete-case analysis, smoking demonstrated a negative effect on clear cell renal cell carcinoma (ccRCC). In contrast, hypertension was associated with higher mortality rates for both ccRCC and non-ccRCC, although this association was not observed in the multiple imputation analysis. Furthermore, a body mass index (BMI) greater than 25 kg/m^2^ was found to significantly reduce overall and cancer-specific mortality in the univariate analysis for ccRCC, but not for non-ccRCC. There was no discernible difference in mortality between current and former smokers.

### 3.2. Overall Mortality

The median follow-up time until overall death for ccRCC was 4.13 (95% CI: 0.8–5.0) years and 2.00 (95% CI: 0.1–5.0) years for non-ccRCC. The five-year overall survival rates for patients with stage III and IV were 47% for ccRCC and 36% for non-ccRCC (Figure 1).

The five-year overall survival rates for patients with primary metastatic disease versus non-metastatic RCC were 16% and 70%, respectively (Figure 2).

In the fully adjusted model, the presence of primary metastasis (HR 2.69 and 2.06), a positive surgical margin (HR 1.95 and 1.53), and a poor performance status (HR 5.93 and 4.73) significantly increased the one-year and five-year overall mortality for ccRCC, respectively (Figure 3).

Similarly, a poor performance status and primary metastasis were associated with an increased risk of death of all causes in patients with non-ccRCC after one year and five years (Figure 4).

### 3.3. Cancer-Specific Mortality

In the fully adjusted model, primary metastasis (HR 16.43 and 3.23), a positive surgical margin (HR 2.03 and 1.43) (with marginal significance for five-year risk of death), and a poor performance status (HR 7.51 and 5.27) significantly increased the one-year and five-year ccRCC-specific mortality, respectively (Figure 5).

Additionally, the presence of primary metastasis and a poor performance status increased non-ccRCC-specific mortality. Meanwhile, the decision taken by the multidisciplinary team was associated with a lower five-year non-ccRCC-specific mortality (Figure 6).

### 3.4. Recurrence

A total of 170 (18%) patients, comprising 147 with ccRCC and 23 with non-ccRCC, experienced recurrence. The median time to recurrence was 2.66 years for ccRCC and 1.44 years for non-ccRCC. In the multivariate analysis, a positive surgical margin was found to significantly increase the risk of recurrence in ccRCC patients, with a hazard ratio of 2.40 (95% confidence interval 1.45–3.94) (Table 2).

### 3.5. Mortality after Recurrence

Out of the 147 ccRCC patients who experienced recurrence, 71 died. The median time to death after recurrence was 2.2 years. The results showed that patients who experienced recurrence more than one year after the initial diagnosis had a lower risk of both overall and cancer-specific mortality compared to patients who experienced recurrence within the first year. Patients who experienced recurrence between 2 and 3 years after the initial diagnosis had the lowest risk of both cancer-specific (HR 0.22; 95% CI: 0.09–0.57) and all-cause mortality (HR 0.22; 95% CI: 0.09–0.54) in comparison to patients who experienced recurrence within the first year after diagnosis (Table 3). On the other hand, patients who received only oncological treatment for recurrent RCC had a higher risk of both overall (HR 5.93; 95% CI: 1.78–19.81) and cancer-specific (HR 8.76; 95% CI: 2.05–37.51) mortality compared to those who received surgical treatment for recurrent RCC.

## 4. Discussion

In this nationwide cohort of Danish stage III and IV RCC patients, we did not find any significant associations between lifestyle factors and worse outcomes. These findings raise the question of the broader implications of lifestyle factors in RCC and whether they may play more nuanced roles than what is reflected in clinical outcomes. However, our study trended towards smoking having a negative effect on ccRCC, although this result was not statistically significant. This is consistent with the wider understanding of smoking’s potential role in carcinogenesis and disease progression in various other cancers [15,16]. Future research might consider larger cohorts or meta-analyses to reach a more definitive conclusion on this. Hypertension was associated with a greater mortality for ccRCC and non-ccRCC in the complete-case analysis, but this relationship was not significant in the multiple imputation (MI) analysis. The effect of hypertension on mortality in RCC has been inconsistent in the literature [5,17]. A BMI greater than 25 kg/m^2^ was associated with a significant reduction in overall and cancer-specific mortality in our univariate analysis for ccRCC, but not in that for non-ccRCC.

Our nationwide Danish cohort contrasts with the Brazilian Oncology Group study [18], which explored obesity and its association with immune checkpoint inhibitors’ (ICI) efficacy. While they found no significant improvement in overall survival among high-BMI patients treated with ICI, our findings demonstrated a significant reduction in overall and cancer-specific mortality in ccRCC patients with a BMI greater than 25 kg/m^2^. Both studies, however, indicate the complexity surrounding the role of BMI in cancer outcomes. Additionally, the inconsistent effects of hypertension on RCC mortality, as observed in our study, highlight the disparities and nuances in patient populations and the need for further investigation.

Consistent with previous research, our study found that a worse performance status was significantly associated with higher cancer-specific and overall mortality in both ccRCC and non-ccRCC patients [19]. However, we did not find a significant association between the presence of symptoms at diagnosis and mortality.

Primary metastasis was strongly associated with an increased mortality, particularly in non-ccRCC, where an eight-fold increase in risk was observed. Patients with primary metastatic non-ccRCC have previously been found to have a significantly shorter time-to-treatment-failure and lower overall survival compared to patients with ccRCC [20].

In this study, the presence of primary metastasis and a poor performance status increased the cancer-specific mortality for both ccRCC and non-ccRCC. When the treatment decision was taken by the MDT, it was associated with a lower five-year cancer-specific mortality in non-ccRCC, but not ccRCC. The relationship between MDT discussion and mortality in cancer patients has been debated, with one study showing no positive benefit for metastatic RCC after an adjustment for clinical factors, and other studies showing no significant relationship for other cancer types [21,22].

Regarding mortality after recurrence, Brookman-May and colleagues found that a recurrence occurring more than a year after primary surgery conferred a 21% lower risk of cancer-specific mortality, similar to our findings [23]. Our results confirmed that receiving both oncological treatment and surgery was associated with a lower mortality risk compared to only receiving oncological treatment, even after an adjustment for factors such as Leibovich score, age, number of metastasis sites, and performance status [24].

### Strengths and Limitations

This study has several strengths, including its nationwide, multicentre design and that the data were extracted from both medical records and registries, enabling the analysis of several important variables. The application of multiple imputations (MI) with a high number of imputations allowed for the efficient use of the cohort. Some differences were noted between the MI and complete-case analysis, but we can assume that values were missing at random (MAR), which makes the MI less biased than the complete-case analysis [25].

Our study, though comprehensive in its approach, does come with some inherent limitations that should be acknowledged when interpreting the results. The retrospective nature of the design, while valuable for understanding historical data patterns, can introduce potential biases, particularly in terms of patient selection and data recording. Consequently, causality cannot be ascertained from our findings. The omission of an adjustment for socioeconomic position might limit the granularity of our results. Socioeconomic factors are known to influence health outcomes, access to care, and adherence to treatments. Thus, not adjusting for them might have potentially masked or skewed some associations. The absence of adjustments for immune checkpoint inhibitor treatments also stands as a significant limitation. Given the rising prominence of these treatments in modern oncology, they can play a pivotal role in determining patient outcomes. Without accounting for them, there could be an inadvertent oversight in understanding the therapeutic implications. Our dataset contained fewer patients with non-ccRCC. This imbalance might lead to the potential of over-adjustment, making some of the analyses less generalizable to the broader non-ccRCC patient population. Lastly, the non-availability of specific biomarkers poses a limitation. In the era of precision medicine, biomarkers can provide in-depth insights into disease prognoses, therapeutic response, and patient outcomes. Their absence in our study might hinder the comprehensive understanding of RCC in the context of the examined lifestyle factors.

## 5. Conclusions

The results of this study suggest that patient-related factors, such as lifestyle, play a less significant role in determining the outcomes of advanced RCC compared to clinical- and disease-related variables. The results also highlight the potential benefits of considering the involvement of multidisciplinary teams during the diagnostic process. Further research is needed to better understand the impact of lifestyle factors on RCC outcomes and explore the differential effects of these factors in terms of cancer stage, particularly in non-clear cell RCC.

## Figures and Tables

**Table 1:** Baseline characteristics of 929 patients diagnosed with stage III or IV renal cancer in Denmark 2014–2016.

Characteristics	Clear Cell N = 768	Non Clear Cell N = 161
Age in years, median (IQR)	67.2 (14.5)	68.5 (12.9)
Female gender, n (%)	254 (33)	50 (31)
BMI, mean (SD)	26.1 (6.2)	25.9 (6.0)
Missing n, (%)	38 (5)	8 (5)
Smoking, n (%)		
Currently	192 (25)	62 (38)
Former	270 (35)	46 (29)
Never	278 (36)	44 (27)
Missing	28 (4)	9 (6)
Hypertension		
Yes	425 (55)	92 (57)
No	343 (45)	69 (43)
Symptoms at time of diagnosis, n (%)	480 (62)	96 (59)
Haematuria	207 (43)	34 (35)
Pain	164 (34)	43 (45)
Weight loss	152 (32)	42 (27)
Other	95 (20)	27 (28)
Performance status, n (%)		
0	393 (51)	67 (41)
1	281 (36)	56 (35)
2	59 (8)	26 (16)
3	27 (3)	9 (6)
4	4 (1)	1 (1)
Missing	4 (1)	2 (1)
T-stage at time of diagnosis, n (%)		
T1	105 (14)	31 (19)
T2	55 (7)	14 (9)
T3	528 (69)	93 (58)
T4	60 (8)	13 (8)
Missing	20 (3)	10 (6)
N stage at time of diagnosis, n (%)		
N0	619 (80)	99 (62)
N1	149 (20)	62 (38)
M stage at time of diagnosis, n (%)		
M0	411 (53)	83 (51)
M1	357 (47)	78 (49)
Size in mm, median (IQR)	79 (40)	70 (64)
Missing, n (%)	23 (3)	6 (4)
Fuhrman grade, n (%)		
1–2	264 (35)	
3–4	437 (57)	
Missing	67 (8)	
Necrosis, n (%)	401 (53)	83 (52)
Leibovich score, n (%)		
0–3	61 (8)	
4–5	261 (34)	
>6	363 (47)	
Missing	83 (11)	
Sarcomatoid, n (%)	100 (13)	24 (15)
Decision taken by MDT, n (%)	377 (49)	73 (46)
Type of surgery, n (%)		
None	151 (19)	59 (36)
Laparoscopy (radical)	323 (42)	35 (22)
Laparoscopy (partial)	30 (4)	14 (9)
Open radical	213 (28)	39 (24)
Open partial	20 (3)	9 (6)
Ablation	10 (1)	3 (2)
Missing	21 (3)	2 (1)
Positive surgical margin, n (%)	75 (10)	20 (12)
Debulking, n (%)	265 (35)	31 (19)
Lymphadenectomy, n (%)	159 (21)	30 (19)
Recurrences, n (%)	147 (36)	23 (28)
Year(s) to first recurrence, median (IQR)	1.6 (2)	0.94 (1.27)
Treatment of recurrent renal cancer, n (%)		
Surgical	25 (17)	6 (26)
Only oncological	68 (46)	8 (35)
Oncological and surgical	28 (19)	4 (17)
No treatment	17 (11)	5 (22)

**Table 2:** Multivariate adjusted hazard ratios (HRs) with corresponding 95% confidence intervals (CIs) for risk of recurrence among 386 clear cell patients diagnosed with clear cell renal cancer without primary metastasis in Denmark 2014–2016.

Exposures	UNIVARIATE	MULTIVARIATE ^A^
		HR (95% CI)
BMI		
>25	1.04 (0.73;1.49)	1.18 (0.81;1.71)
Smoking		
Currently/previously	0.93 (0.66;1.30)	1.02 (0.73;1.45)
Symptoms		
Yes	1.72 (1.21;2.46) *	1.27 (0.88;1.83)
Decision taken by MDT		
Yes	1.67 (1.19;2.36) *	1.33 (0.92;1.92)
Hypertension		
Yes	1.06 (0.76;1.49)	1.18 (0.82;1.70)
Positive surgical margin ^B^		
Yes	2.42 (1.51;3.89) *	2.40 (1.45;3.94) *
Performance status		
1–2	0.76 (0.52;1.11)	0.79 (0.53;1.17)
3–4	1.28 (0.18;9.19)	2.34 (0.31;17.63)

^A^ Adjusted for age, gender, Leibovich score, sarcomatoid, smoking, hypertension, decision taken by MDT, and lymphadenectomy; ^B^ Also adjusted for partial or radical nephrectomy; * *p* < 0.05.

**Table 3:** Multivariate adjusted overall mortality and cancer-specific hazard ratios (HRs) with corresponding 95% confidence intervals (CIs) among 141 patients diagnosed with recurrent clear cell renal cancer in Denmark 2014–2016.

Exposures	OVERALL MORTALITY ^A^	CANCER-SPECIFIC MORTALITY ^A^
	HR (95% CI)	HR (95% CI)
Time to recurrence (Reference: 0–1 years)		
1–2 years	0.40 (0.22;0.70) *	0.44 (0.24;0.79) *
2–3 years	0.22 (0.09;0.54) *	0.22 (0.09;0.57) *
3+ years	0.46 (0.20;1.05)	0.40 (0.16;1.01)
Treatment of recurrence ^B^ (Reference: only surgical treatment)		
Only oncological	5.93 (1.78;19.81) *	8.76 (2.05;37.51) *
Surgical and oncological	3.01 (0.80;11.33)	4.11 (0.86;19.58)
No treatment	3.49 (0.85;14.33)	3.69 (0.65;20.93)

^A^ Adjusted for age, gender, Leibovich score, performance status, and hypertension; ^B^ Additionally adjusted for whether recurrence was single or multiple site; * *p* < 0.05.

## Data Availability

All data generated or analysed during this study are included in this published article and its supplementary tables.

## Supplementary Materials

The following supporting information can be downloaded at: https://www.mdpi.com/article/10.3390/cancers15184488/s1, Table S1: Complete-case analysis of multivariate adjusted mortality hazard ratios (HRs) with corresponding 95% confidence intervals (CIs) for overall risk of death within 1 year or 5 years among 646 patients diagnosed with clear cell renal cancer in Denmark 2014–2016.; Table S2: Complete-case analysis of multivariate adjusted mortality hazard ratios (HRs) with corresponding 95% confidence intervals (CIs) for overall risk of death within 1 year or 5 years among 141 patients diagnosed with non-clear cell renal cancer in Denmark 2014–2016. Table S3: Complete-case analysis of multivariate adjusted mortality hazard ratios (HRs) with corresponding 95% confidence intervals (CIs) for 1-year and 5-year risk of cancer specific death among 661 patients diagnosed with clear cell renal cancer in Denmark 2014–2016. Table S4: Complete-case analysis of multivariate adjusted mortality hazard ratios (HRs) with corresponding 95% confidence intervals (CIs) for 1-year and 5-year risk of cancer specific death among 141 patients diagnosed with non-clear cell renal cancer in Denmark 2014–2016. Table S5: MI analysis of multivariate adjusted mortality hazard ratios (HRs) with corresponding 95% confidence intervals (CIs) for overall risk of death within 1 year or 5 years among 746 patients diagnosed with clear cell renal cancer in Denmark 2014–2016. Table S6: MI analysis of multivariate adjusted mortality hazard ratios (HRs) with corresponding 95% confidence intervals (CIs) for overall risk of death within 1 year or 5 years among 159 patients diagnosed with non-clear cell renal cancer in Denmark 2014–2016. Table S7: MI analysis of multivariate adjusted mortality hazard ratios (HRs) with corresponding 95% confidence intervals (CIs) for 1-year and 5-year risk of cancer specific death among 746 patients diagnosed with clear cell renal cancer in Denmark 2014–2016. Table S8: MI analysis of multivariate adjusted mortality hazard ratios (HRs) with corresponding 95% confidence intervals (CIs) for 1-year and 5-year risk of cancer specific death among 159 patients diagnosed with non-clear cell renal cancer in Denmark 2014–2016.

## Abbreviations

Renal cell carcinoma	RCC
Examine the effect of patient-related and clinical factors on overall and cancer-specific mortality	RQ1
Examine the effect of patient-related and clinical factors on recurrence	RQ2
Investigate the impact of time to recurrence and the treatment of recurrences on overall and cancer-specific mortality	RQ3
Danish Renal Cancer Database	DaRenCa
Body Mass Index	BMI
Multidisciplinary team	MDT
Clear cell RCC	ccRCC
Non-clear cell RCC	non-ccRCC
Hazard ratios	HR
Confidence intervals	CI
Multiple imputations	MI
Missing at random	MAR
