# Supplementary material for: Lifestyle and Clinical Factors in a Nationwide Stage III and IV Renal Cell Carcinoma Study
Source: Cancers (Basel). 2023 Sep 9;15(18):4488. doi: 10.3390/cancers15184488 (PMC10526437; doi:10.3390/cancers15184488)
Supplement: Supplementary file 1 [file cancers-15-04488-s001.zip › cancers-2561120-supplementary.pdf]

Supplementary Table S1

Complete-case analysis of multivariate adjusted mortality hazard ratios (HRs) with corresponding 95% confidence intervals (CIs) for **overall risk of death within 1 year or 5 years among 646 patients** diagnosed with **clear cell renal cancer** in Denmark 2014-2016.

Exposures	UNIVARIATE	MULTIVARIATE [A]
		HR (95% CI)
**BMI**		
>25 (1 year)	0.52 (0.36;0.77)*	0.68 (0.45;1.02)
>25 (5 year)	0.70 (0.56;0.88)*	0.87 (0.69;1.10)
**Smoking**		
Current/previous (1 year)	1.39 (0.92;2.10)	0.87 (0.56;1.35)
Current/previous (5 year)	1.35 (1.06;1.72)*	1.05 (0.82;1.36)
**Symptoms**		
Yes (1 year)	1.23 (0.82;1.84)	0.72 (0.46;1.12)
Yes (5 year)	1.41 (1.10;1.80)*	0.98 (0.76;1.27)
**Primary metastasis**		
Yes (1 year)	8.95 (5.32;15.05)*	3.77 (1.94;7.33)*
Yes (5 year)	4.90 (3.84;6.24)*	2.85 (2.06;3.96)*
**Decision taken in MDT**		
Yes (1 year)	2.25 (1.50;3.37)*	1.00 (0.65;1.54)
Yes (5 year)	2.04 (1.62;2.58)*	1.11 (0.86;1.43)
**Hypertension**		
Yes (1 year)	1.16 (0.79;1.72)	1.21 (0.80;1.85)
Yes (5 year)	1.30 (1.03;1.63)*	1.30 (1.02;1.66)*
**Positive surgical margin [B]**		
Yes (1 year)	2.90 (1.71;4.90)*	1.74 (1.01;2.98)*
Yes (5 year)	1.91 (1.37;2.65)*	1.37 (0.97;1.92)
**Performance status**		
1-2 (1 year)	3.04 (1.97;4.69)*	2.27 (1.43;3.61)*
3-4 (1 year)	18.25(9.50;35.04)*	8.57(3.90;18.85)*
1-2 (5 year)	1.87 (1.48;2.36)*	1.41 (1.09;1.82)*
3-4 (5 year)	9.88 (5.93;16.47)*	5.09 (2.85;9.10)*

[A] Adjusted for age, gender, Leibovich score, sarcomatoid, smoking, hypertension, performance status, decision taken in MDT, debulking, lymphadenectomy

[B] Also adjusted for partial or radical nephrectomy

*p<0.05

Supplementary table S2

<u>Complete-case analysis</u> of multivariate adjusted mortality hazard ratios (HRs) with corresponding 95% confidence intervals (CIs) for **overall risk of death within 1 year or 5 years** among 141 patients diagnosed with **<u>non-clear cell renal cancer</u>** in Denmark 2014-2016.

**Exposures**	**UNIVARIATE**	**MULTIVARIATE[A]**
		HR (95% CI)
**BMI**		
>25 (1 year)	0.88 (0.50;1.56)	1.21 (0.66;2.22)
>25 (5 year)	0.77 (0.50;1.18)	0.87 (0.55;1.40)
**Smoking**		
Current/previous (1 year)	2.24 (1.17;4.29)*	2.26 (0.97;5.25)
Current/previous (5 year)	1.56 (0.98;2.48)	1.97 (1.09;3.57)*
**Symptoms**		
Yes (1 year)	1.75 (0.97;3.17)	0.97 (0.45;2.08)
Yes (5 year)	1.45 (0.94;2.25)	0.97 (0.57;1.65)
**Primary metastasis**		
Yes (1 year)	14.46 (6.14;34.01)*	4.48 (1.46;13.76)*
Yes (5 year)	10.93 (6.54;18.27)*	5.08 (2.41;10.73)*
**Decision taken in MDT**		
Yes (1 year)	2.11 (1.21;3.70)*	0.82 (0.43;1.58)
Yes (5 year)	1.63 (1.07;2.49)*	0.64 (0.39;1.04)
**Hypertension**		
Yes (1 year)	2.00 (1.10;3.66)*	2.27 (1.14;4.50)*
Yes (5 year)	1.32 (0.86;2.04)	1.67 (1.00;2.77)*
**Positive surgical margin [B]**		
Yes (1 year)	2.93 (1.15;7.45)*	0.83 (0.28;2.44)
Yes (5 year)	2.55 (1.32;4.93)*	0.64 (0.28;1.47)
**Performance status**		
1-2 (1 year)	6.08 (2.71;13.65)*	3.73 (1.45;9.57)*
3-4 (1 year)	16.63 (5.78;47.84)*	6.81 (1.95;23.75)*
1-2 (5 year)	3.77 (2.29;6.23)*	2.30 (1.26;4.18)*
3-4 (5 year)	13.12 (5.58;30.83)*	4.55 (1.70;12.19)*

[A] Adjusted for age, T-stage, N-stage, M-stage, size, hypertension, performance status, debulking
[B] Also adjusted for partial or radical nephrectomy

*p<0.05

Supplementary table S3

Complete-case analysis of multivariate adjusted mortality hazard ratios (HRs) with corresponding 95% confidence intervals (CIs) for **1-year & 5 year risk of cancer specific death among 661** patients diagnosed with **clear cell renal cancer** in Denmark 2014-2016.

Exposures	UNIVARIATE	MULTIVARIATE[A]
		HR (95% CI)
**BMI**		
>25 (1 year)	0.49 (0.32;0.76)*	0.67 (0.43;1.05)
>25 (5 year)	0.66 (0.51;0.86)*	0.85 (0.65;1.11)
**Smoking**		
Current/previous (1 year)	1.25 (0.80;1.95)	0.71 (0.44;1.16)
Current/previous (5 year)	1.28 (0.98;1.68)	0.93 (0.70;1.24)
**Symptoms**		
Yes (1 year)	1.49 (0.94;2.38)	0.77 (0.46;1.28)
Yes (5 year)	1.79 (1.34;2.39)*	1.08 (0.79;1.46)
**Primary metastasis**		
Yes (1 year)	47.91(15.14;151.67)*	19.27(5.45;68.14)*
Yes (5 year)	8.90 (6.52;12.14)*	4.58 (3.06;6.85)*
**Decision taken in MDT**		
Yes (1 year)	2.18 (1.40;3.41)*	0.83 (0.51;1.32)
Yes (5 year)	2.41 (1.84;3.14)*	1.09 (0.82;1.45)
**Hypertension**		
Yes (1 year)	1.01 (0.66;1.56)	1.20 (0.76;1.91)
Yes (5 year)	1.18 (0.91;1.53)	1.30 (0.99;1.72)
**Positive surgical margin [B]**		
Yes (1 year)	3.34 (1.86;6.00)*	1.74 (0.95;3.17)
Yes (5 year)	2.05 (1.42;2.96)*	1.26 (0.86;1.84)
**Performance status**		
1-2 (1 year)	3.35 (2.04;5.49)*	2.37 (1.40;4.02)*
3-4 (1 year)	25.24 (12.73;50.07)*	11.47(4.91;26.80)*
1-2 (5 year)	1.83 (1.40;2.38)*	1.34 (1.01;1.79)*
3-4 (5 year)	10.87 (6.29;18.80)*	5.55 (2.95;10.48)*

[A] Adjusted for age, gender, Leibovich score, sarcomatoid, smoking, hypertension, performance status, decision taken in MDT, debulking, lymphadenectomy
[B] Also adjusted for partial or radical nephrectomy

*p<0.05

Supplementary table S4

Complete-case analysis of multivariate adjusted mortality hazard ratios (HRs) with corresponding 95% confidence intervals (CIs) for **1-year & 5 year risk of cancer specific death among 141 patients** diagnosed with **non-clear cell renal cancer** in Denmark 2014-2016.

Exposures	UNIVARIATE	MULTIVARIATE [A]
		HR (95% CI)
**BMI**		
>25 (1 year)	0.89 (0.50;1.59)	1.30 (0.70;2.42)
>25 (5 year)	0.79 (0.50;1.26)	0.96 (0.58;1.59)
**Smoking**		
Current/previous (1 year)	2.05 (1.06;3.96)*	1.99 (0.86;4.62)
Current/previous (5 year)	1.76 (1.06;2.95)*	2.63 (1.34;5.17)*
**Symptoms**		
Yes (1 year)	1.95 (1.04;3.63)*	0.93 (0.42;2.06)
Yes (5 year)	1.85 (1.13;3.04)	1.11 (0.61;2.02)
**Primary metastasis**		
Yes (1 year)	20.99 (7.52;58.61)*	8.21 (2.26;29.87)*
Yes (5 year)	16.86 (8.96;31.73)*	7.68 (3.21;18.38)*
**Decision taken in MDT**		
Yes (1 year)	2.25 (1.26;4.01)*	0.78 (0.40;1.53)
Yes (5 year)	1.81 (1.14;2.86)*	0.60 (0.36;1.03)
**Hypertension**		
Yes (1 year)	2.02 (1.09;3.77)*	2.04 (1.01;4.14)*
Yes (5 year)	1.32 (0.83;2.11)	1.66 (0.94;2.90)
**Positive surgical margin [B]**		
Yes (1 year)	2.93 (1.15;7.44)*	0.75 (0.25;2.23)
Yes (5 year)	2.56 (1.25;5.25)*	0.46 (0.19;1.09)
**Performance status**		
1-2 (1 year)	5.70 (2.53;12.86)*	3.80 (1.39;10.33)*
3-4 (1 year)	14.09 (4.69;42.29)*	6.53 (1.72;24.76)*
1-2 (5 year)	3.90 (2.24;6.77)*	2.64 (1.29;5.42)*
3-4 (5 year)	12.28 (4.91;30.67)*	5.15 (1.73;15.37)*

[A] Adjusted for age, T-stage, N-stage, M-stage, size of tumor, sarcomatoid, hypertension, performance status, debulking
[B] Also adjusted for partial or radical nephrectomy

*p<0.05

Supplementary table S5

MI-analysis of multivariate adjusted mortality hazard ratios (HRs) with corresponding 95% confidence intervals (CIs) for **overall risk of death** within 1 year or 5 years among 746 patients diagnosed with clear cell renal cancer in Denmark 2014-2016.

**Exposures**	**UNIVARIATE**	**MULTIVARIATE** [A]
	HR (95 % CI)	HR (95 % CI)
**BMI**		
>25 (1 year)	0.66 (0.48;0.90)*	0.85 (0.61;1.20)
>25 (5 year)	0.73 (0.60;0.90)*	0.96 (0.78;1.19)
**Smoking**		
Current/previous (1 year)	1.44 (1.02;2.03)*	1.02 (0.71;1.45)
Current/previous (5 year)	1.35 (1.09;1.67)*	1.04 (0.83;1.30)
**Symptoms**		
Yes (1 year)	1.33 (0.96;1.84)	0.93 (0.66;1.31)
Yes (5 year)	1.35 (1.10;1.67)*	1.03 (0.83;1.28)
**Primary metastasis**		
Yes (1 year)	9.78 (6.19;15.47)*	2.69 (1.38;5.24)*
Yes (5 year)	5.35 (4.28;6.67)*	2.06 (1.48;2.88)*
**Decision taken in MDT**		
Yes (1 year)	2.13 (1.55;2.93)*	0.92 (0.66;1.29)
Yes (5 year)	2.02 (1.65;2.47)*	1.09 (0.88;1.35)
**Hypertension**		
Yes (1 year)	1.03 (0.75;139)	1.11 (0.80;1.54)
Yes (5 year)	1.17 (0.96;1.43)	1.13 (0.92;1.40)
**Positive surgical margin** [B]		
Yes (1 year)	2.70 (1.60;4.55)*	1.95 (1.14;3.31)*
Yes (5 year)	1.91 (1.38;2.65)*	1.53 (1.09;2.14)*
**Performance status**		
1-2 (1 year)	3.08 (2.14;4.42)*	1.91 (1.29;2.82)*
3-4 (1 year)	16.91(10.25;27.88)*	5.93(3.34;10.51)*
1-2 (5 year)	2.28 (1.85;2.82)*	1.49 (1.18;1.88)*
3-4 (5 year)	11.35 (7.59;16.98)*	4.73 (3.02;7.40)*

[A] Adjusted for age, gender, Leibovich score, sarcomatoid, smoking, hypertension, performance status, decision taken in MDT, debulking, lymphadenectomy
[B] Also adjusted for partial or radical nephrectomy

*p<0.05

Supplementary table S6

MI-analysis of multivariate adjusted mortality hazard ratios (HRs) with corresponding 95% confidence intervals (CIs) for **overall risk of death** within 1 year or 5 years among 159 patients diagnosed with non-clear cell renal cancer in Denmark 2014-2016.

Exposures	UNIVARIATE	MULTIVARIATE [A]
	HR (95 CI %)	HR (95 % CI)
**BMI**		
>25 (1 year)	0.82 (0.48;1.40)	1.19 (0.67;2.11)
>25 (5 year)	0.78 (0.51;1.18)	0.96 (0.61;1.52)
**Smoking**		
Current/previous (1 year)	1.88 (1.06;43.35)*	1.77 (0.82;3.80)
Current/previous (5 year)	1.38 (0.91;2.10)	1.62 (0.91;2.89)
**Symptoms**		
Yes (1 year)	1.72 (1.00;2.97)*	1.18 (0.61;2.29)
Yes (5 year)	1.45 (0.96;2.18)	1.22 (0.74;2.01)
**Primary metastasis**		
Yes (1 year)	13.06 (5.92;28.83)*	3.68(1.29;10.48)*
Yes (5 year)	10.14 (6.18;16.65)*	4.39 (2.08;9.28)*
**Decision taken in MDT**		
Yes (1 year)	1.88 (1.12;3.14)*	0.80 (0.44;1.47)
Yes (5 year)	1.65 (1.11;2.46)*	0.64 (0.40;1.04)
**Hypertension**		
Yes (1 year)	1.52 (0.89;2.58)	1.75 (0.95;3.25)
Yes (5 year)	1.23 (0.82;1.84)	1.39 (0.85;2.27)
**Positive surgical margin [B]**		
Yes (1 year)	2.84 (1.12;7.21)*	0.84 (0.30;2.39)
Yes (5 year)	2.67 (1.37;5.20)*	0.74 (0.33;1.65)
**Performance status**		
1-2 (1 year)	6.99 (3.14;15.55)*	3.93 (1.60;9.63)*
3-4 (1 year)	17.33 (6.41;46.84)*	8.42(2.67;26.52)*
1-2 (5 year)	3.82 (2.36;6.18)*	2.35 (1.36;4.06)*
3-4 (5 year)	11.77 (5.39;25.70)*	5.72(2.36;13.88)*

[A] Adjusted for age, T-stage, N-stage, M-stage, size of tumour, hypertension, performance status, debulking
[B] Also adjusted for partial or radical nephrectomy

*p<0.05

Supplementary table S7

MI-analysis of multivariate adjusted mortality hazard ratios (HRs) with corresponding 95% confidence intervals (CIs) for 1-year & 5 year risk of **cancer specific death** among 746 patients diagnosed with clear cell renal cancer in Denmark 2014-2016.

**Exposures**	**UNIVARIATE**	**MULTIVARIATE** [A]
	HR (95 % CI)	HR (95 % CI)
**BMI**		
>25 (1 year)	0.61 (0.43;0.86)*	0.84(0.58;1.22)
>25 (5 year)	0.69 (0.55;0.87)*	0.94(0.74;1.20)
**Smoking**		
Current/previous (1 year)	1.28 (0.89;1.84)	0.89(0.60;1.30)
Current/previous (5 year)	1.25 (0.99;1.59)	0.91 (0.71;1.16)
**Symptoms**		
Yes (1 year)	1.67 (1.15;2.43)*	1.09(0.74;1.61)
Yes (5 year)	1.69 (1.32;2.16)*	1.15(0.89;1.48)
**Primary metastasis**		
Yes (1 year)	64.97(20.69;204.00)*	16.43(4.57;59.07)*
Yes (5 year)	10.11 (7.53;13.56)*	3.23 (2.14;4.87)*
**Decision taken in MDT**		
Yes (1 year)	2.06 (1.46;2.91)*	0.74 (0.51;1.07)
Yes (5 year)	2.33 (1.85;2.93)*	1.06 (0.83;1.35)
**Hypertension**		
Yes (1 year)	0.90 (0.65;1.26)	1.05 (0.74;1.51)
Yes (5 year)	1.04 (0.84;1.30)	1.10 (0.87;1.38)
**Positive surgical margin** [B]		
Yes (1 year)	3.13 (1.75;5.60)*	2.03 (1.12;3.67)*
Yes (5 year)	2.05 (1.42;2.96)*	1.43 (0.98;2.09)
**Performance status**		
1-2 (1 year)	3.44 (2.29;5.18)*	2.02 (1.30;3.13)*
3-4 (1 year)	21.74 (12.73;37.12)*	7.51 (4.10;13.75)*
1-2 (5 year)	2.33 (1.84;2.96)*	1.48 (1.14;1.92)*
3-4 (5 year)	12.72 (8.27;19.56)*	5.27 (3.26;8.52)*

[A] Adjusted for age, gender, Leibovich score, sarcomatoid, smoking, hypertension, performance status, decision taken in MDT, debulking, lymphadenectomy

[B] Also adjusted for partial or radical nephrectomy

*p<0.05

Supplementary table S8

MI-analysis of multivariate adjusted mortality hazard ratios (HRs) with corresponding 95% confidence intervals (CIs) for 1-year & 5 year risk of **cancer specific death** among 159 patients diagnosed with non-clear cell renal cancer in Denmark 2014-2016.

**Exposures**	**UNIVARIATE**	**MULTIVARIATE [A]**
	HR (CI 95 %)	HR (95 % CI)
**BMI**		
>25 (1 year)	0.86 (0.50;1.50)	1.29(0.72;2.32)
>25 (5 year)	0.82 (0.52;1.28)	1.07(0.66;1.75)
**Smoking**		
Current/previous (1 year)	1.68 (0.94;3.00)	1.48(0.69;3.19)
Current/previous (5 year)	1.48 (0.93;2.36)	1.85(0.98;3.48)
**Symptoms**		
Yes (1 year)	1.83 (1.03;3.23)*	1.05 (0.53;2.11)
Yes (5 year)	1.71 (1.08;2.71)*	1.20 (0.67;2.12)
**Primary metastasis**		
Yes (1 year)	22.14 (7.99;61.39)*	8.14(2.29;28.98)*
Yes (5 year)	18.96 (9.80;36.66)*	9.28(3.66;23.52)*
**Decision taken in MDT**		
Yes (1 year)	2.06 (1.20;3.52)*	0.75 (0.40;1.42)
Yes (5 year)	1.85 (1.19;2.86)*	0.59 (0.35;0.99)*
**Hypertension**		
Yes (1 year)	1.59 (0.91;2.76)	1.63 (0.86;3.06)
Yes (5 year)	1.23 (0.79;1.91)	1.34 (0.79;2.28)
**Positive surgical margin [B]**		
Yes (1 year)	2.83 (1.11;7.19)*	0.72 (0.24;2.06)
Yes (5 year)	2.73 (1.32;5.67)*	0.48 (0.21;1.14)
**Performance status**		
1-2 (1 year)	6.48 (2.91;14.44)*	4.03(1.57;10.32)*
3-4 (1 year)	13.07 (4.57;37.42)*	7.57(2.20;26.02)*
1-2 (5 year)	4.09 (2.39;6.99)*	2.63 (1.38;4.99)*
3-4 (5 year)	10.27 (4.32;24.37)*	5.62(2.09;15.15)*

[A]Model 1: Adjusted for age, T-stage, N-stage, M-stage, size of tumour, sarcomatoid, hypertension, performance status, debulking
[B] Also adjusted for partial or radical nephrectomy

*p<0.05
